# A case of adrenal metastasis of hepatocellular carcinoma diagnosed by endoscopic ultrasound‐guided fine‐needle aspiration

**DOI:** 10.1002/deo2.362

**Published:** 2024-04-10

**Authors:** Tsuyoshi Ueda, Shinji Oe, Akitoshi Yoneda, Yudai Koya, Satoru Nebuya, Koichiro Miyagawa, Yuichi Honma, Michihiko Shibata, Shohei Shimajiri, Masaru Harada

**Affiliations:** ^1^ Third Department of Internal Medicine School of Medicine University of Occupational and Environmental Health Fukuoka Japan; ^2^ Department of Pathology and Cell Biology School of Medicine University of Occupational and Environmental Health Fukuoka Japan

**Keywords:** adrenal metastasis, adrenal tumor, EUS‐FNA, hepatocellular carcinoma, lung adenocarcinoma

## Abstract

An 82‐year‐old man had been treated for lung adenocarcinoma and hepatocellular carcinoma (HCC). Contrast‐enhanced computed tomography examination showed swelling of the left adrenal gland, suggesting metastasis of lung adenocarcinoma, HCC, or primary adrenal tumor. Endoscopic ultrasound‐guided fine‐needle aspiration (EUS‐FNA) was performed for the pathological diagnosis, and adrenal metastasis of HCC was diagnosed. No notable complications due to EUS‐FNA were found. There have been reports of adrenal metastasis due to various cancers, but there are few reports that can confirm the diagnosis of adrenal metastasis of HCC using EUS‐FNA. Adrenal metastasis of HCC is not a rare condition, but it may be difficult to diagnose in the case of multiple cancer complications. We experienced a case in which EUS‐FNA was useful for the diagnosis of adrenal metastasis of HCC.

## INTRODUCTION

Endoscopic ultrasound (EUS) is the widely used medical imaging method for viewing abdominal diseases. EUS‐guided fine needle aspiration (EUS‐FNA) is used for a diagnostic procedure to investigate pathological findings.^1^ Many tumors can develop adrenal metastasis, and lung cancer is the most common cancer. The incidence of adrenal metastasis in patients with hepatocellular carcinoma (HCC) is reportedly 6.9%–19.1%.^2^ Although some cases of adrenal metastasis from lung carcinoma have been reported, there were only two cases of adrenal metastasis of HCC diagnosed by EUS‐FNA.^3^, ^4^ We report here a case of adrenal metastasis from HCC diagnosed by EUS‐FNA and able to treat HCC.

## CASE REPORT

An 82‐year‐old man was suffered from lung cancer. As a family history, his brother has a history of malignant tumors. His medical history includes surgery for bladder cancer at the age of 47. Drinking history was opportunity drinking and smoking history was 20 cigarettes per day for 60 years.

**FIGURE 1:**
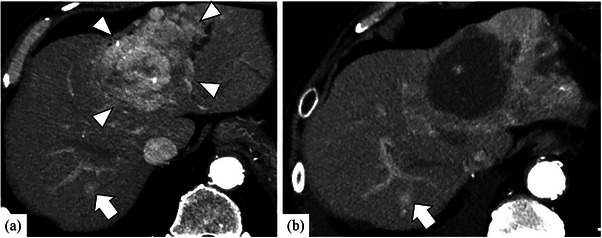
(a) Computed tomography during the arterial phase showed a tumor with 7 cm in diameter (white arrowheads) in liver segment 4. Computed tomography showed the high‐density area in liver segment 7 (white arrow). (b) Computed tomography was performed after the first transcatheter arterial chemoembolization, and the tumor in liver segment 4 could not be detected. Furthermore, it revealed that a tumor with a 1 cm diameter was detected in liver segment 7 as a variable hepatocellular carcinoma (white arrow).

Computed tomography (CT) revealed that right upper lung cancer was stage IIIA. And a 7 cm diameter liver tumor was detected in liver segment 4. Furthermore, CT showed the high‐density area in liver segment 7 (Figure 1a). He received surgery for stage IIIA right upper lobe adenocarcinoma without neoadjuvant chemotherapy.

**FIGURE 2:**
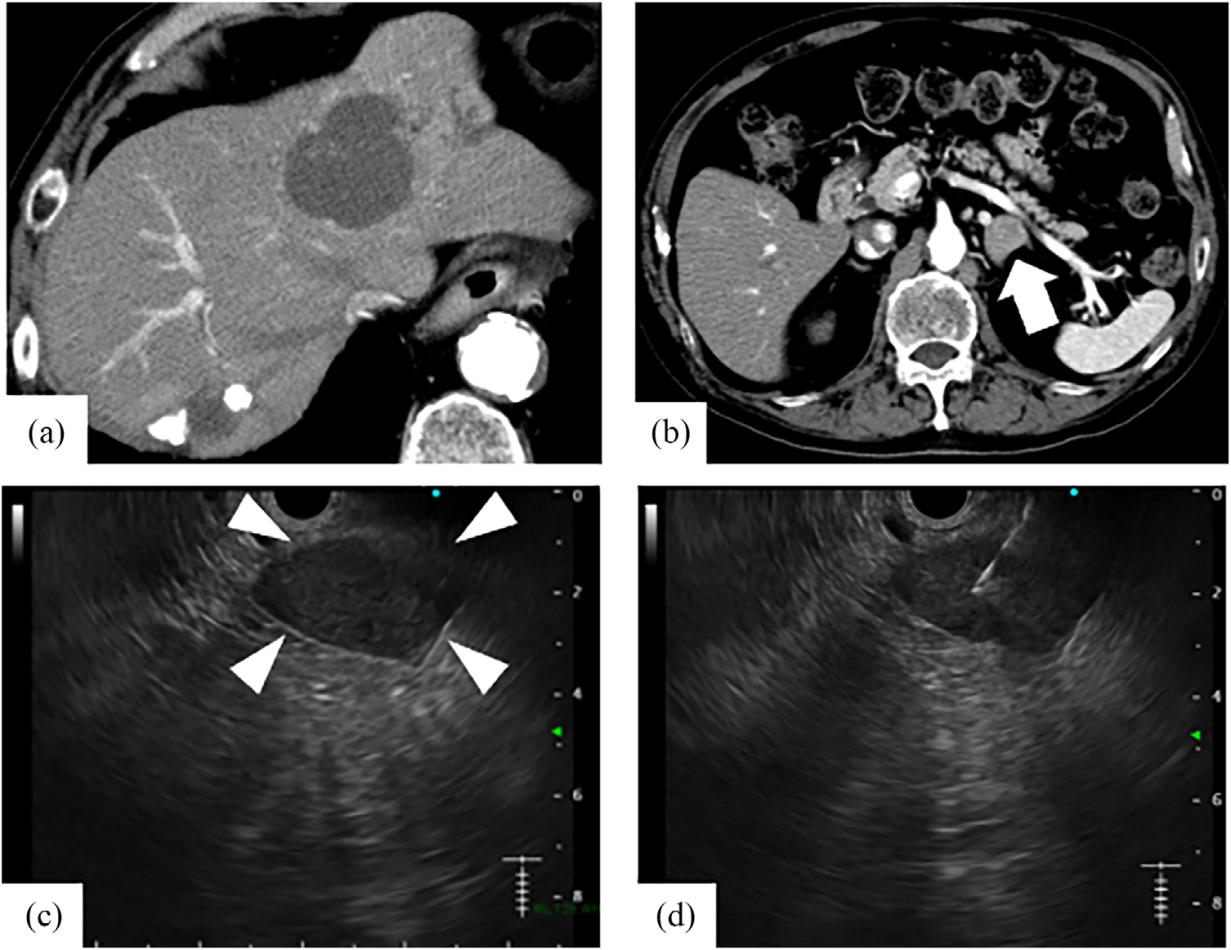
(a, b) Computed tomography showed a tumor with 23 mm in diameter at the left adrenal gland, suggesting the possibility of an adrenal metastasis from lung adenocarcinoma, hepatocellular carcinoma, or a primary adrenal gland tumor (white arrow). And liver tumors were not detected in liver segments 4 and 7 on computed tomography. (c, d) Endoscopic ultrasound shows a hypoechoic mass in the left adrenal gland (white arrowheads). We performed endoscopic ultrasound‐guided fine‐needle aspiration of the left adrenal gland using a 25‐gauge needle.

**FIGURE 3:**
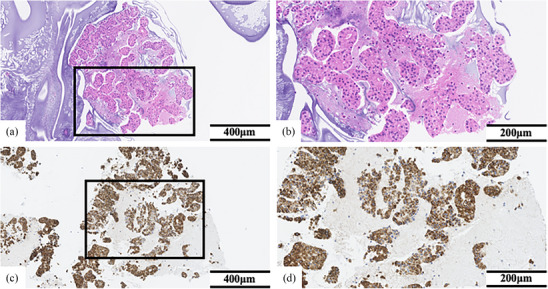
Fine‐needle aspiration cytology images of different adrenal gland lesions. A tissue obtained using a 25‐gauge needle was enough large for the histological evaluation. (a) The cells with round nucleolus with small nuclear and acidophilic cytoplasm are seen in a cord‐like, glandular duct‐like structure with hematoxylin and eosin staining (scale bar = 400 µm). (b, d) Higher magnification of the boxed area each shown in (a) and (c) (scale bar = 200 µm). The specimen is enlarged with the boxed area shown in (a), which can diagnosis easily of hepatocellular carcinoma (scale bar = 200 µm). (c) Immunohistochemical staining for hepatocyte paraffin 1 is positive (scale bar = 400 µm). The boxed area shown enlarged in (d) showing with immunostaining is positive for hepatocyte paraffin 1 (scale bar = 200 µm).

The tumor in liver segment 4 was diagnosed with HCC of stage III by fine needle aspiration biopsy, and transcatheter arterial chemoembolization (TACE) was performed twice two months. Since the high‐density area with CT in liver segment 7 was not as typical as HCC, it stayed under observation. A month after performing TACE twice, CT revealed that HCC in liver segment 4 almost disappeared, but a high‐density area in liver segment 7 was diagnosed with new HCC (Figure 1b). So, TACE was performed again for HCC in liver segment 7. Three months after performing TACE three times, CT revealed a mass lesion measuring 23 mm in diameter at the left adrenal gland, and the primary HCC was not in the liver (Figure 2a,b). It suggested the possibility of an adrenal metastasis from lung adenocarcinoma, HCC, or a primary adrenal gland tumor. Because the serum levels of tumor markers were within the normal range, we couldn't accurately diagnose the tumor. Therefore, we decided to perform EUS‐FNA. A physical examination showed no abnormality on admission. According to the guidelines of pheochromocytoma, if the serum and urine levels of catecholamine were within the normal range as in this case (Table 1), 123I‐metaiodobenzylguanidine (MIBG) scintigraphy was not always necessary. Therefore, MIBG scintigraphy was not performed in this case. EUS revealed a well‐defined hypoechoic mass in the left adrenal gland, and the lesion displayed a poor Doppler sign. EUS‐FNA was performed using a 25‐gauge core needle (Acquire; Boston Scientific Co.; Figure 2c,d). The puncture was performed twice, and there were no complications associated with EUS‐FNA including hemorrhage. The day after the EUS‐FNA, the serum and urine levels of catecholamine were within the normal range (Table S1). Pathological findings showed that tumor cells with eosinophilic cytoplasm and circular nuclear including small nucleolus formed strands and gland‐like structures. In the immunohistochemical staining, hepatocyte‐paraffin‐1 positive cells were observed in the tumor (Figure 3). Fine needle biopsy of the liver tumor revealed a well to moderately differentiated HCC. HCC is composed of trabecular and pseudoglandular patterns with hyperchromatic nuclei and eosinophilic granules resembling hepatocytes. These pathological findings were like that from EUS‐FNA of the adrenal gland tumor. Because of these findings, we diagnosed the patients as HCC stage IVB with left adrenal metastasis. HCC was present only in the left adrenal gland, and the patient had good liver function (Child‐Pugh A). Because he refused to receive the excision of the adrenal tumor, he received TACE for left adrenal metastasis from HCC. However, he had a poor therapeutic response to TACE and started treatment with lenvatinib. After treatment with lenvatinib for three months, follow‐up CT revealed that the tumor size increased from 23 to 52mm in diameter at the left adrenal gland. After about two weeks, he developed hypothyroidism. He had been medicated with levothyroxine, but his general condition worsened. It was difficult to continue chemotherapy due to the decline in performance status, so palliative care was started. After about a month, he died because of deterioration of general conditions

**TABLE 1:** The blood test data before the endoscopic ultrasound guided‐fine needle aspiration.

Peripheral blood			Biochemistry			Serology			Viral marker		
WBC	6300	/µL	Total protein	7.0	g/dL	CRP	0.4	mg/dL	HBsAg	Negative	
Eosinophil	3.0	%	Albumin	3.7	g/dL				HBcAb	0.1	S/CO
Basophil	0.9	%	Total bilirubin	0.6	mg/dL	**Endocrine**			HCVAb	Negative	
Lymphocyte	14.1	%	TG	189	mg/dL	COR	9.1	µg/dL			
Monocyte	8.4	%	HDL‐Cho	46	mg/dL	PRA	1.2	ng/mL/h	**Urinalysis (24 h urine)**		
Neutrophil	73.6	%	LDL‐Cho	134	mg/dL	PAC	140	pg/mL	Urinary MN	7.3	µg/day
RBC	418 × 10^4^	/µL	T‐Chol	223	mg/dL	DHEA‐S	185	ng/mL	Urinary NMN	153.8	µg/day
Hemoglobin	13.4	g/dL	CK	64	U/L	Dopamine	1072.2				
Platelet	26.1 × 10^4^	/µL	AST	19	U/L						
			ALT	13	U/L	**Coagulation**					
			ALP	143	U/L	PT‐%	93	%			
			LDH	193	U/L	PT‐INR	1.05				
			BUN	14	mg/dL						
			Creatinine	1.07	mg/dL	**Tumor makers**					
			Glucose	207	mg/dL	CEA	3.8	ng/mL			
			HbA1c (NGSP)	6.2	%	SLX	25.3	U/mL			
			Na	135	mmol/L	AFP	6.9	ng/dL			
			K	4.7	mmol/L	PIVKA‐II	13	mAU/mL			
			Cl	100	mmol/L						

Abbreviations: AFP, alpha‐fetoprotein; ALP, alkaline phosphatase; ALT, alanine aminotransferase; AST, aspartate aminotransferase; BUN, blood urea nitrogen; CEA, carcinoembryonic antigen; CK, creatine kinase; Cl, chlorine; COR, cortisol; CRP, C‐reactive protein; DEHA‐S, dehydroepiandrosterone sulfate; HbA1c, hemoglobin A1c; HBcAb, hepatitis B core antibody; HBsAg, hepatitis B surface antigen; HCVAb, hepatitis C antibody; HDL‐Cho, high‐density lipoprotein cholesterol; K, potassium; LDH, lactic dehydrogenase; Na, sodium; PAC, plasma aldosterone concentration; PIVKA‐II, protein induced by vitamin K absence or antagonist II; PRA, plasma renin activity; PT, prothrombin time; RBC, red blood cell; SLX, sialyl Lewis X; TG, triglyceride; Urinary MN, urinary metanephrine; Urinary NMN, urinary normetanephrine; WBC, white blood cell.

## DISCUSSION

Abdominal ultrasonography, CT, and magnetic resonance imaging are indispensable for diagnosing cancer. In situations such as double cancer or the existence of inflammation, these tests alone can make it difficult to diagnose the metastasis. In addition, tissue examination is also required for definitive diagnosis of cancer, but the method of tissue examination is limited for retroperitoneal organs such as the adrenal gland. In the gastrointestinal field, EUS is a valuable method for diagnosing cancer. If it can be visualized by EUS, it is possible to perform histological examination by EUS‐FNA. Since the EUS‐FNA was reported by Vilmann et al. in 1992,^1^ it has become possible to collect samples of deep organs such as the adrenal gland relatively safely and easily with the progress of technology. Martinez et al. performed EUS‐FNA on 95 swollen adrenal tumors and reported favorable results that 92% could be achieved with a definitive diagnosis and no complications were observed.^5^

The probability that an adrenal tumor is a metastatic tumor is estimated at about 2% in non‐cancer‐bearing patients and about 30–70% in cancer‐bearing patients.^6^ The adrenal gland has abundant blood flow and is easily metastasized by various kinds of cancers. Malignant melanoma, thyroid cancer, renal cell carcinoma, pancreatic cancer, colon cancer, and lung cancer are often the primary lesions. Of the 398 autopsy cases of HCC from 1988 to 2012, 156 (39.1%) had extrahepatic metastases, 74.5% had lung metastases, 24.8% had bone metastases, 19.1% had adrenal metastases, 17.1 had local lymph nodes.^7^ There are few reports that adrenal metastasis of HCC compared to lung cancer. There are only two reports that diagnosed adrenal metastasis of HCC by EUS‐FNA.^3^, ^4^

The diagnostic accuracy of adrenal metastatic tumors by EUS‐FNA has been shown in one retrospective study of 150 patients of lung cancer, in which six (4%) had CT and five (3.3%) had PET‐CT showing metastases in one or both adrenal glands and EUS‐FNA was performed in 11 (7.3%) patients, with a diagnosis of adrenal metastases in four patients.^8^ In this report, the diagnostic accuracy of adrenal metastatic tumors is 96% by CT, 97% by PET‐CT, and 100% by EUS‐FNA. In this case, it was difficult to distinguish whether it was a primary adrenal tumor or a metastatic tumor because of the history of treatment for lung adenocarcinoma and HCC. A left adrenal tumor could be visualized by EUS, and FNA was performed and pathological examination was performed. By comparing the histopathological findings of previous HCC diagnoses, adrenal tumors could be diagnosed as metastasis of HCC. If we did not perform EUS‐FNA for a left adrenal tumor, the alternative methods of EUS‐FNA were percutaneous ultrasound‐guided fine‐needle aspiration, and laparoscopic biopsy et al. Some reports suggest that the right adrenal gland can be sampled with EUS‐FNA by a duodenal approach. However, EUS‐FNA of the right adrenal gland requires us to twist the scope, and more likely to result in perforation. Therefore, the success rate of the EUS‐FNA of the right adrenal gland is not high, and it is not a safe examination.^9^

There are reports that EUS‐FNA did not cause any complications for adrenal tumors diagnosed as pheochromocytoma by pathological results, however, one study described hypertensive crisis occurred as the complication.^10^ Therefore, blood tests should be performed before EUS‐FNA to check for pheochromocytoma comorbidity and adequate informed consent should be performed for pheochromocytoma‐associated crises. It was considered that using a 25‐gauge needle reduced the risk of bleeding and a potential pheochromocytoma using fine‐needle biopsy needles increased the amount of tissue that could be collected.

The advantages of EUS‐FNA compared to percutaneous biopsy are that there is no radiation exposure or contrast administration, and real‐time echo‐guided puncture is possible. It is important to perform a whole‐body search in advance, not to mention drawing and puncture skills, to determine the indication and purpose of EUS‐FNA, and to perform it appropriately.

## CONFLICT OF INTEREST STATEMENT

None.

## Supporting information

Table S1.The blood test data after the endoscopic ultrasound guided‐fine needle aspiration.
